# A gastrointestinal stromal tumor with acute bleeding: Management and nursing

**DOI:** 10.1097/MD.0000000000009874

**Published:** 2018-03-02

**Authors:** Xiuju Shi, Shuxia Yu, Fenyan Wang, Qi Zhao, Hongwei Xu, Bin Li

**Affiliations:** a Department of Gastroenterology, Shandong Provincial Hospital Affiliated to Shandong University; b Department of Gastroenterology, Qianfo Mountain Hospital, Jinan, China.

**Keywords:** acute bleeding, gastrointestinal stromal tumor, polidocanol, sclerotherapy

## Abstract

**Rationale:**

Gastrointestinal stromal tumors (GISTs) are the most common mesenchymal tumors involving the gastrointestinal tract. A small percentage of GISTs may cause acute gastrointestinal bleeding, which requires urgent surgical intervention.

**Patient concerns:**

In this case report, we present a 62-year-old male patient with who was hospitalized due to acute bleeding.

**Diagnoses:**

The patient was diagnosed as GIST with low risk.

**Interventions:**

The patient was treated endoscopically with polidocanol sclerotherapy.

**Outcomes:**

The mass was removed completely, and the patient was discharged at day 9 after operation.

**Lessons:**

This case indicates that GIST can present as massive upper gastrointestinal bleeding and urgent endoscopic sclerotherapy can be life-saving. The endoscopical intervention may be a good alternative for emergency.

## Abstract 2

Supplemental Digital Content is available in the text

## 1 Introduction

Gastrointestinal stromal tumors (GISTs) are the most common mesenchymal tumors involving the gastrointestinal tract. Since GISTs usually present with nonspecific clinical manifestation, they may be found incidentally at endoscopy or surgery for an unrelated reason, or even found at autopsy.^[1,2]^ A small percentage of GISTs may cause acute symptoms, such as GI bleeding, which is requiring urgent surgical, endoscopical intervention or transcatheter arterial embolization.^[3–6]^ In this case report, we present a patient with GIST who was hospitalized due to acute bleeding and then treated endoscopically with polidocanol sclerotherapy.

## 2 Case presentation

A 62-year-old male was admitted to Shandong Provincial Hospital due to hematemesis and melena for 4 days. The patient also reported an 11-year history of mitral inadequacy, without chest pain or shortness of breath.

The patient had experienced melena for twice during the previous 4 years. Neither esophagogastroduodenal endoscopy (EGD) nor colonoscopy was conclusive.

**Figure 1:**
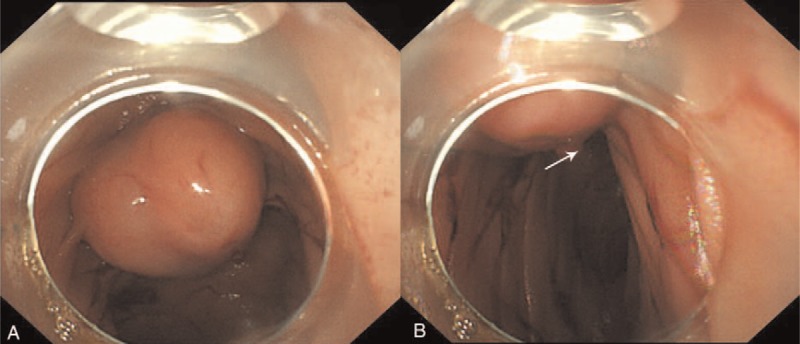
The gastrointestinal stromal tumor. (A) The lesion locating at the proximal loops of the jejunum. (B) An adherent clot on the top of the lesion (the arrow points).

The patient was severely anemic with an initial hemoglobin concentration of 3.9 g/dL. A complete physical examination revealed no pertinent findings. After blood transfusing with 4 U red blood cell and 500 mL serum, the patient underwent urgent EGD, which demonstrated curious dark blood in the second portion of the duodenum. We thus assumed that the bleeding lesion might locate at the duodenum or at the proximal loops of the jejunum. Colonoscopy was herein used and showed a 2.0 cm × 2.5 cm submucosal mass at the proximal loops of the jejunum. There was an ulcerative pit on the top of the mass, within which was an adherent clot (Fig. 1). Fresh blood was extruded at the center of the pit when the patient felt nausea. The patient was then suspected as stromal tumor with active bleeding.

Considering that the patient still had massive bleeding and the blood pressure kept going down, urgent endoscopical sclerotherapy was performed. A 5 mL test dose of polidocanol was administered by injecting into the mass body with a 23 G injection needle, followed by 2 mL aliquots were injected circumferentially surrounding the center of the pit. The total volume of polidocanol was 15 mL. The sclerotherapy was done till the surface of the mass bulged and the active bleeding terminated (Supplementary Video). Restriction from oral intake was required for 24 hours after the sclerotherapy procedure. Red blood cell (2 U) was transfused in the operation. The hemoglobin level was determined again 6 hours after the treatment, and it increased to 8.1 g/dL, indicating the bleeding stopped successfully. The patient felt much better than before and was then transferred to the department of surgery. The hemoglobin level increased to 10.8 g/dL 5 days after the sclerotherapy.

**Figure 2:**
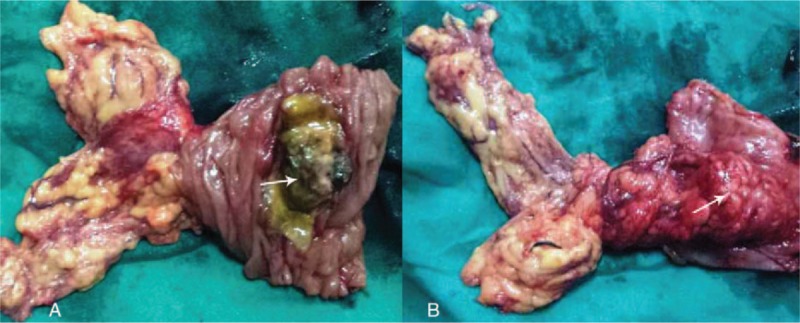
Surgical specimen. (A) The GIST in the intestine. (B) The tumor outside the intestine (the arrow points).

**Figure 3:**
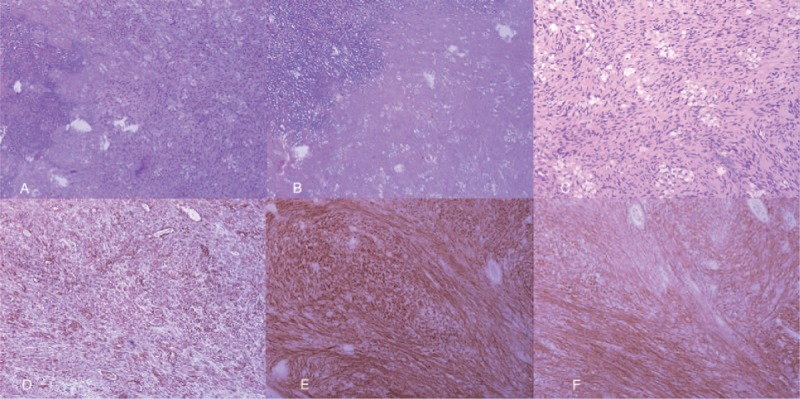
The pathological examinations. (A) The lesion (hematoxylin–eosin staining, 100×). (B) The necrosis area of the lesion (hematoxylin–eosin staining, 100×). (C) Non necrotic area of the lesion (hematoxylin–eosin staining, 200×). (D) Positive CD34 staining (immunohistochemisty staining, 100×). (E) Positive CD117 staining (immunohistochemisty staining, 200×). (F) Positive Dog-1 staining (immunohistochemisty staining, 200×).

Seven days after the procedure, the patient received the surgery to remove the mass completely. The operation revealed a mass, locating at the jejunum, approximately 20 cm to Treiz ligament, without bleeding, which had already invaded to the greater omentum (Fig. 2). The immunohistochemical studies showed positive staining of tumor cells for CD34, CD117, and Dog-1, and negative staining for smooth muscle actin (SMA) and S-100 protein (Fig. 3). The mitotic activity was 1 mitosis/50 high-power field (HPF). These findings strongly supported a diagnosis of low risk of GIST. The patient was discharged at day 9 after operation.

Written informed consent was obtained from the patient, and the consent procedure and study protocol were approved by the Medical Institutional Ethical Committee of the Provincial Hospital Affiliated to Shandong University.

## 3 Discussion and conclusion

GIST was first reported by Mazur and Clark as a separate entity from gastrointestinal smooth muscle tumors in 1983,^[7]^ with an estimated unadjusted incidence of around 1/100,000/year.^[8]^ The most common site for a GIST is the stomach (52%), followed by the small intestine (25%).^[2]^ Most patients with GISTs present with nonspecific symptoms, such as early satiety and bloating.^[9]^ GISTs are often discovered incidentally during endoscopic procedures. Several major GIST-related symptoms are bleeding, abdominal pain, abdominal mass, and obstruction.

The surgical resection and radiologic embolization are the most effective methods in the management of GISTs with acute bleeding. At present, surgical resection remains the mainstay approach in treating the patients with localized, nonmetastatic GIST.^[1,10,11]^ It can both stop the bleeding and resect the lesion. As for this case, the bleeding was so fierce at endoscopy and the patient, who have a history of 4 years chronic bleeding, might not have the chance of surgical resection. Since the mass located at the jejunum and drifted away from the organ, the lesion was difficult to be held by the cap. Argon plasma coagulation or electrical coagulation could not achieve a satisfied outcome. Due to the large size of the mass, clips could not close the lesion either. Endoscopic ligation treatment are theoretically feasible, however with high freeness from the organ, the operation is extremely difficult. As for this case, the bleeding is so fierce and the patient might not have the chance of surgical resection. Sclerotherapy dates back at least 1 century,^[12]^ which is generally used for vices, vascular malformations and hemorrhoids.^[13,14]^ Endoscopic sclerotherapy is particularly benefit for patients with esophageal variceal bleeding.^[15,16]^ Normally polidocanol is injected into the vessels, which makes them shrink. The degeneration and necrosis of the tissue can be extensively seen after the sclerotherapy.

Of noted, the procedure of sclerotherapy is relatively simple. Nowadays, sclerotherapy is mainly used for the treatment of esophagus vices. To the best of our knowledge, this is the first case report of a GIST with acute bleeding that was successfully managed endoscopically with polidocanol sclerotherapy. This case indicates that GIST can present as massive upper gastrointestinal bleeding and urgent endoscopic sclerotherapy can be life-saving. The endoscopical intervention may be a good alternative for emergency.

The appropriate nursing during the operation is significant. The patient is left lateral position. The trachea cannula can be used to protect the trachea before the operation if condition allowed. As for this case, we observed bleeding at endoscopy, so the trachea cannula was not available. The nurse must observe intensively on the changes of consciousness, oxygen saturation, blood pressure, heart rate, and respiration movement of patients in the process of operation. In addition, during the emergency, we followed the way injecting curing agency. We injected the polidocanol in multiple point inside the tumor, firstly down side, then lateral, and finally upside. When the mucous membrane became raised, the bleeding was stopped. The injection should avoided delayed bleeding or perforation. The nurse should pay attention to control the injection speed of polidocanol not to be too fast, and the injection dose must be controlled to be less than 2 mL per injection and 15 mL in total. Since the arterial bleeding is comparatively fierce, blood transfusing is highly effective and will earn us some operating time. The nurse also should supervise the patients to abrosia, stay in bed, and observe intensively on the occurrence of hematemesis, melena. Forty-eight hours after the surgery, the enteral nutrition with liquid diet is first choice. This patient observed no recurrence or metastasis, indicating GIST treated endoscopically with polidocanol sclerotherapy is feasible.

## Supplementary Material

Supplemental Digital Content
