# A gastrointestinal stromal tumor with acute bleeding: Management and nursing: Erratum

**DOI:** 10.1097/MD.0000000000010492

**Published:** 2018-04-13

In the article, “A gastrointestinal stromal tumor with acute bleeding: Management and nursing”^[1]^, which appeared in Volume 97, Issue 9 of *Medicine*, Dr. Fengyan Wang's name appeared incorrectly as Fenyan Wang.
